# Redescription of the poorly known planktonic copepod *Pontellopsis lubbockii* (Giesbrecht, 1889) (Pontellidae) from the Eastern Tropical Pacific with a key to species

**DOI:** 10.3897/zookeys.234.3933

**Published:** 2012-10-29

**Authors:** Eduardo Suárez-Morales, Eva Kozak

**Affiliations:** 1 El Colegio de la Frontera Sur (ECOSUR), Av. Centenario Km 5.5, A.P. 424, Chetumal, Quintana Roo 70014, Mexico; 2 Centro Universitario de la Costa Sur (CUCSUR), Universidad de Guadalajara. San Patricio Melaque, Jalisco, Mexico

**Keywords:** Zooplankton, Mexican Pacific, taxonomy of copepods, biodiversity, pelagic crustaceans

## Abstract

During a survey of the epipelagic zooplankton carried out off the coast of the Mexican states of Jalisco and Colima, in the Eastern Tropical Pacific, female and male specimens of the poorly known calanoid copepod *Pontellopsis lubbockii* (Giesbrecht, 1889) were collected. Because previous descriptions and illustrations are largely incomplete and have caused some taxonomical confusion, this species is fully redescribed from specimens from the Mexican Pacific. The species has some characters that have been overlooked, but those related to the female genital double-somite are the most striking, it has two conical dorsal protuberances and a long ventral spiniform process unique of this species. The mouthparts of this species have not been hitherto described and figured, the flexible terminal setae of legs 3 and 4 is noteworthy. The male general morphology agrees in general with previous data, but new details of the leg 5 and geniculate antennule are added. Its mouthparts, with strong, serrate setae on the maxillae and maxillules, and a strong mandibular edge, suggest that this is a predator form. A dichotomous key for the identification of males and females of the species of *Pontellopsis* known from the Eastern Tropical Pacific is included.

## Introduction

The genus *Pontellopsis* Brady, 1883 currently contains up to 33 species (Boxshall and Halsey 2004; Razouls et al. 2012; Walter and Boxshall 2012). As other members of the family Pontellidae, species of *Pontellopsis* are usually recorded in surface waters (0–10 m) of tropical and warm temperate latitudes (Othman and Toda 2006). In general, pontellids are regarded as good indicators of water masses (Sherman 1963, 1964; Matsuo and Marumo 1982; Hernández-Trujillo 1989). Because of their morphological complexity and variability (Fleminger 1956, 1967b, 1975; Silas and Pillai 1973), their taxonomy is still in flux, partly caused by incomplete descriptions that have raised taxonomic confusion in different regions (Pillai 1977; Jeong et al. 2009). Therefore, in some instances, it is necessary to revise and redescribe species following upgraded modern standards in order to facilitate the identification of these species and related forms (Mulyadi 2002; El-Sherbiny and Ueda 2008). One of these poorly defined pontellid species is *Pontellopsis lubbockii* (Giesbrecht, 1889), whose original description and subsequent illustrations by Giesbrecht (1893) Wilson (1950), and Pillai (1977) are limited and lacking in detail. Several important characters of this species have been omitted, not only details of the taxonomically relevant characters, but of the mouthparts and legs 1-4, which still remain undescribed. Besides the occurrence of this species at the type locality off Columbia (Giesbrecht 1889), Wilson (1950) reported this species from the Eastern Pacific; the identity of some of Wilson’s specimens were revised by Pillai (1977), who noticed some inconsistencies both in its identification and in the records related to this species. Wilson (1950) identified and labeled female pontellids from the off Sri Lanka, in the South Pacific as *Pontellopsis lubbockii* but Pillai (1977) noticed that these were in fact specimens of *Pontellopsis krämeri* (Giesbrecht, 1896); in the same sample he found also copepodites of *Pontella* sp. and of *Labidocera acuta* (Dana, 1849).

*Pontellopsis lubbockii* has been relatively rarely taken and is known as a neritic equatorial species endemic to the Gulf of California and adjacent areas of the Eastern Tropical Pacific (ETP) (Brinton et al. 1986, Suárez-Morales and Gasca 1998) and extending to Ecuador (Pillai 1977). Overall, the pontellid copepod fauna of the area south of the influence of the California Current and off the Mexican and Central American coasts of the Pacific is still poorly known. Previous regional surveys by Alameda-De la Mora (1980), Álvarez-Cadena (1985), Suárez-Morales and Gasca (1989), Morales-Ramírez (2001), Fernández-Álamo et al. (2000), Álvarez-Silva et al. (2003), and Morales-Ramírez and Suárez-Morales (2009) include only one species of *Pontellopsis* in this area of the ETP and *Pontellopsis lubbockii* was not recorded. In some instances this could be a result of misidentifications or the rarity of the species. In this work we report and redescribe this poorly known pontellid based on female and male specimens collected during a series of zooplankton surveys carried out off the central Mexican Pacific coast. We also provide comments on the morphology of the mouthparts and a key for the identification of the males and females of the species of *Pontellopsis* recorded in this region.

## Material and methods

The zooplankton samples analyzed were obtained at twelve sites sampled during 27 months between December 1995 and December 1998 on board the R/V BIP-V and “León Marino”. Samples were collected at night time by oblique, semicircular trawls at different depths (10- 115 m) with a Bongo net (0.5 mm mesh size). The zooplankton samples were fixed and preserved with 4% formaldehyde buffered with sodium borate. Copepods were sorted from the original samples and transferred to 70% ethanol with a drop of glycerine for further analysis. Voucher specimens were deposited in the Zooplankton collection of El Colegio de la Frontera Sur, Chetumal, Mexico (ECO-CHZ).

## Systematics

### Order Calanoida Sars, 1903

Family Pontellidae Dana, 1853

*Pontellopsis* Brady, 1883

*Monops* Lubbock, 1853

*Pseudomonops* Claus, 1892

*Pontellopsis lubbockii* (Giesbrecht, 1889)

#### Monops lubbockii

Giesbrecht, 1899

http://species-id.net/wiki/Monops_lubbockii

**Figure 1.**
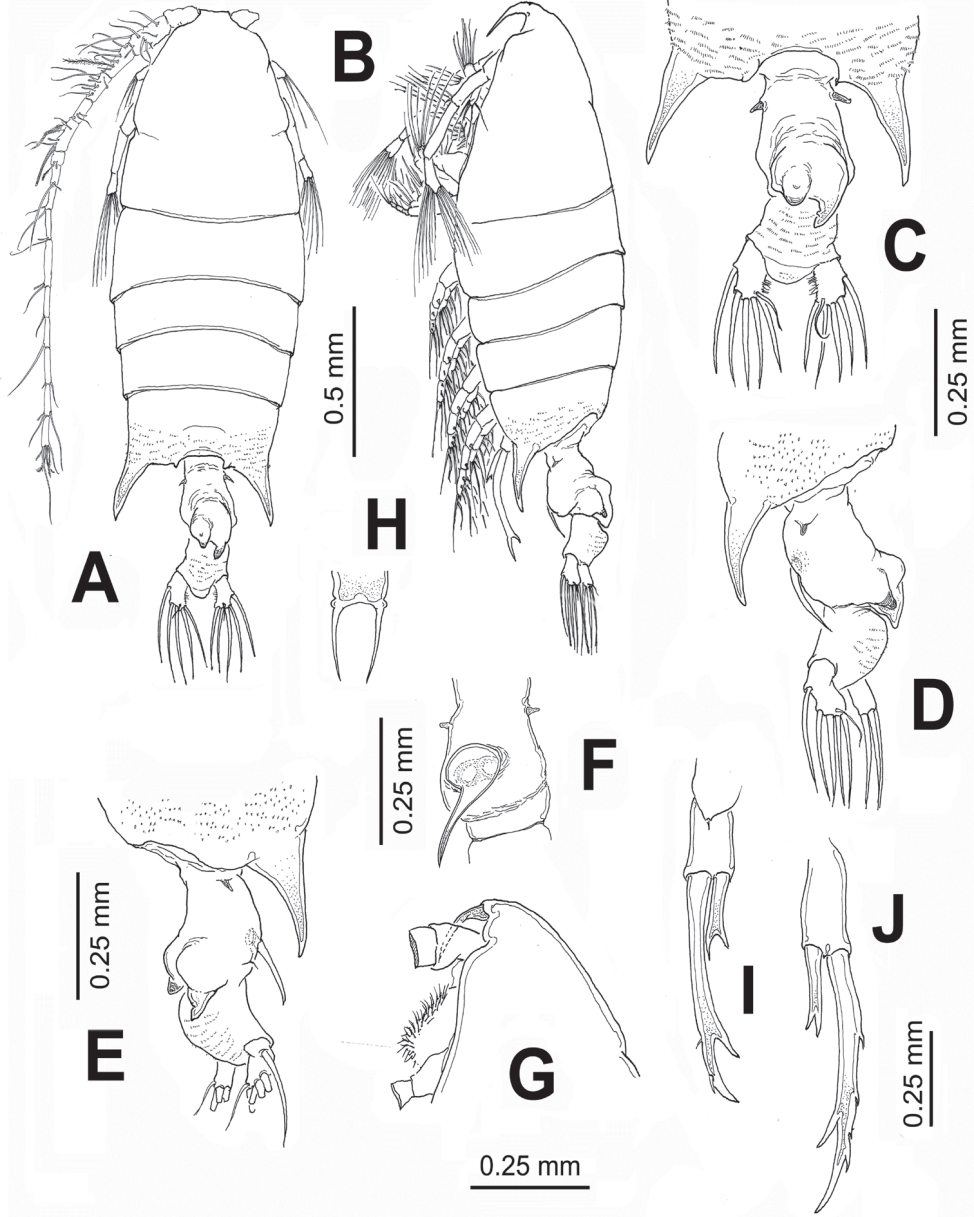
*Pontellopsis lubbockii* (Giesbrecht) from the Mexican Pacific. Adult female **A** habitus, dorsal view **B** same, lateral view **C** urosome showing details of dorsal processes of genital double-somite, ventral view **D** same, left lateral view **E** same, right lateral view **F** genital double-somite, ventral view **G** cephalic section, lateral view **H** rostrum, ventral view **I** right leg 5 **J** left leg 5.

Figs 1 [Fig F2] [Fig F3] [Fig F4] 5

##### Type locality.

Eastern Tropical Pacific (3–6°N, 80–82° W), about 400 km west of the coasts of Colombia and 320 km south of the Panama coast.

##### Material examined.

Two adult females from the central Pacific of Mexico, 14 December 2010, 19.171°N, 104.912°W, coll. E. Kozak and C. Franco-Gordo, specimens undissected, vial deposited at El Colegio de la Frontera Sur, Chetumal, Mexico (ECO-CHZ-08957). One adult male, same date, site, and collector; specimen dissected, semi-permanent slides sealed with Entellan® (ECO-CHZ-08958). One adult male, 25 October, 2011, same site and collector; specimen dissected in slides sealed with Entellan® (ECO-CHZ-08959). One adult female, 24 October, 2011, 19.171°N, 104.912°W, coll. C. Franco-Gordo; specimen undissected, ethanol-preserved, vial (ECO-CHZ-08960). One adult male, 25 September, 1997, 19.033°N, 104.674°W, coll. C. Franco-Gordo; specimens undissected, ethanol-preserved, vial deposited in ECOSUR (ECO-CHZ-08961). One adult female from Californian coast, 7 October, 1904, 30.67°N, 119.59°W, Albatross cruise, Eastern Pacific Expedition, ethanol-preserved, identified by A. Fleminger (USNM-109384). One adult female from off Ecuador, South Pacific Ocean, 8 November, 1928, 01.531°N, 82.273°W, Carnegie Institution of Washington, ethanol-preserved (USNM-80382), previously examined by P. Pillai.

##### Female.

Body length of females range between: 2.09 and 2.17 mm (average 2.13 mm, *n*=5), measured from anterior cephalosome to posterior border of anal somite. Cephalosome robust, widest at level of fully separated first pedigerous somite. Pedigerous somites 4 and 5 fused; posterior corners of fifth pedigerous somite strongly developed, forming large spine-like processes (Fig. 1A, B). Processes straight, posteriorly directed, reaching about halfway along urosome. Cephalosome with rounded forehead, dorsal lenses absent. Rostrum bifid, with long, slender rostral filaments, gap between rostral rami wide (Fig. 1H), in lateral view reaching halfway of second antennular segment (Fig. 1G). Urosome with two segments: genital double somite and anal somite. Genital double-somite representing about 55% of urosome length, excluding caudal rami; somite strongly asymmetrical, with pair of dorsal protuberances arising from distal margin of somite (Fig. 1C, D). In dorsal view, right protuberance subtriangular, curved, posteriorly directed, reaching about half way along anal somite. Left process smaller, also posteriorly directed rounded tapering distally into strongly chitinized bulb-like process (Fig. 1C). Proximal margin of somite bearing lateral spine-like process on each margin, slightly asymmetrical, right one being longer. Ventral surface of genital double somite swollen, with sickle-shaped process arising anterior to genital operculum, posteriorly directed (Fig. 1D, E, F). Anal somite subrectangular, about 1.5 times wider than long, with rounded distomedial process between insertion points of caudal rami. Dorsal surface of anal somite swollen in lateral view, ornamented with rows of minute spinules. Caudal rami weakly asymmetrical, left ramus slightly larger than right, both rami bearing 6 setae: 1 inner, 3 terminal, 1 outer setae plus short, slender dorsal seta.

**Figure 2. F2:**
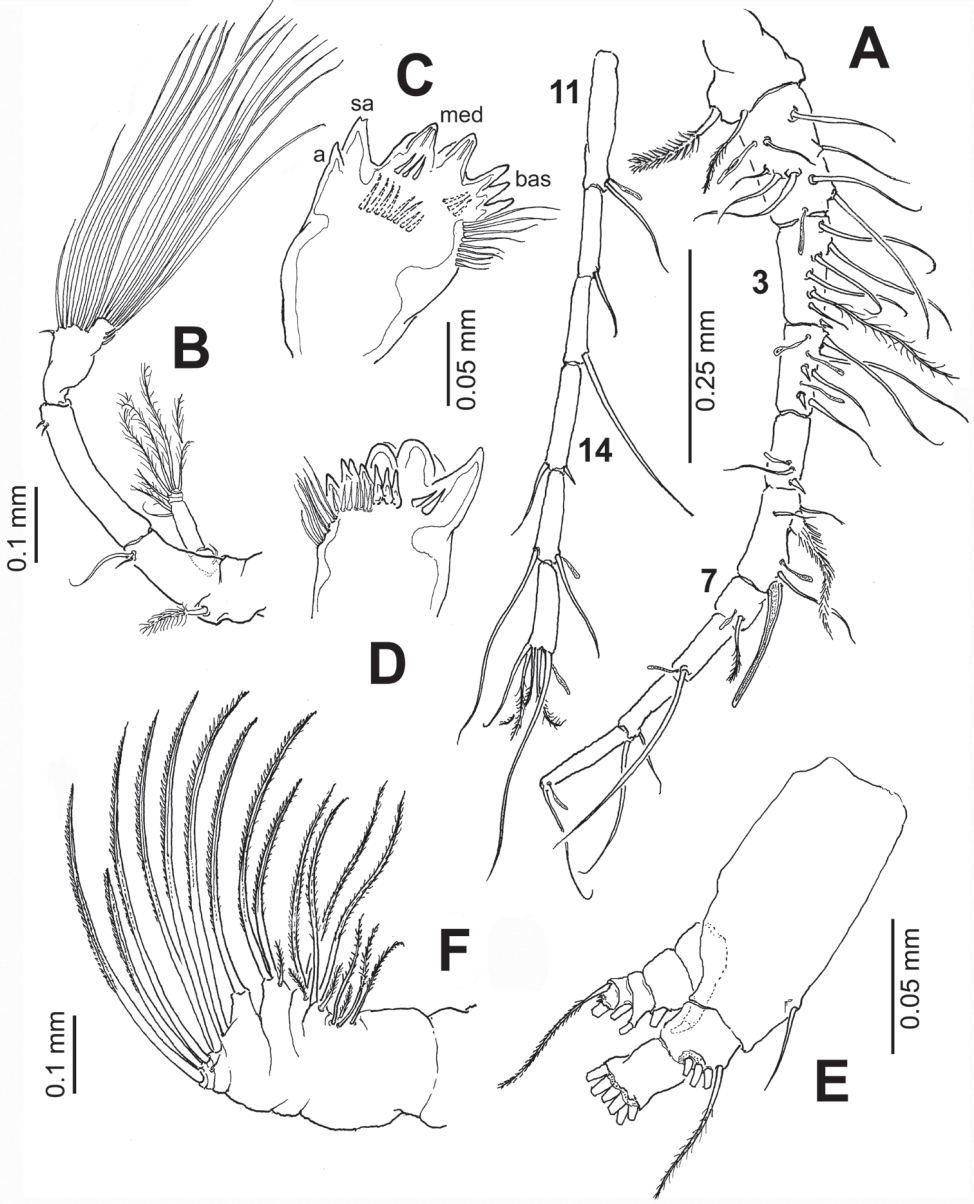
*Pontellopsis lubbockii* (Giesbrecht) from the Mexican Pacific. Adult female **A** antennule (in two sections) **B** antenna **C** mandible edge showing dentition, apical (**a**), subapical (**sa**), medial (**med**), and basal (**bas**) teeth **D** same, another view **E** mandibular palp **F** maxilla.

Antennules (Fig. 2A) symmetrical, 16-segmented. Segments armed as follows (Arabic numbers= setae; Roman numerals= spines, aes=aesthetascs): 1 (I-III) (1), 2 (IV-VII) (9+aes), 3 (VIII-X) (6,I+aes), 4 (XI-XIII) (4,II+3aes), 5 (XIV) (1,I+aes), 6 (XV-XVI) (4,I+ 2aes), 7(XVII) (1+aes), 8(XVIII) (1+aes), 9 (XIX) (1+aes), 10 (XX) (1+aes), 11 (XXI) (1+aes), 12 (XXII) (1), 13 (XXIII) (1), 14 (XXIV) (1,I), 15 (XXV) (2+aes), 16 (XXVI-XXVIII) (4+aes). Larger and longer setae on segments 2, 4, 7, 8, and 13. Modified, wide-based heavily setulated seta proximally inserted on segment 6; same segment with distally blunt, strongly chitinized spine reaching about 2/3 of way along succeeding segment 7 (Fig. 2A).

Antenna (Fig. 2B) biramous: coxa with short plumose distal seta. Basis and first endopodal segment separated, basis bearing 2 setae, one short, one long. First endopodal segment elongate, armed with two small subdistal setae. Second endopodal segment with 9 and 7 setae on proximal and distal lobes, respectively; distal lobe armed with basal outer row of spinules; exopod 6-segmented, setation formula 1, 2, 1, 1, 1,2.

Mandible (Figs 2C–E) with wide, heavily chitinized gnathobase; mandibular palp biramous, basipod robust, subrectangular, armed with inner basipodal seta. Endopod 2-segmented, first segment armed with 3 long and one short setae; second segment with 6 terminal setae. Exopod 5-segmented, setal formula as: 1, 1,1,1,2. Mandibular distal edge bearing 7 teeth: from ventral margin dentition includes one apical (a), one subapical (sa), two compound medial (med), and three basal (bas) (see Fig. 2C); medial teeth with rounded edges. Clusters of long and short spinules on base of medial teeth; dorsal end of gnathobase with tight row of setae.

**Figure 3. F3:**
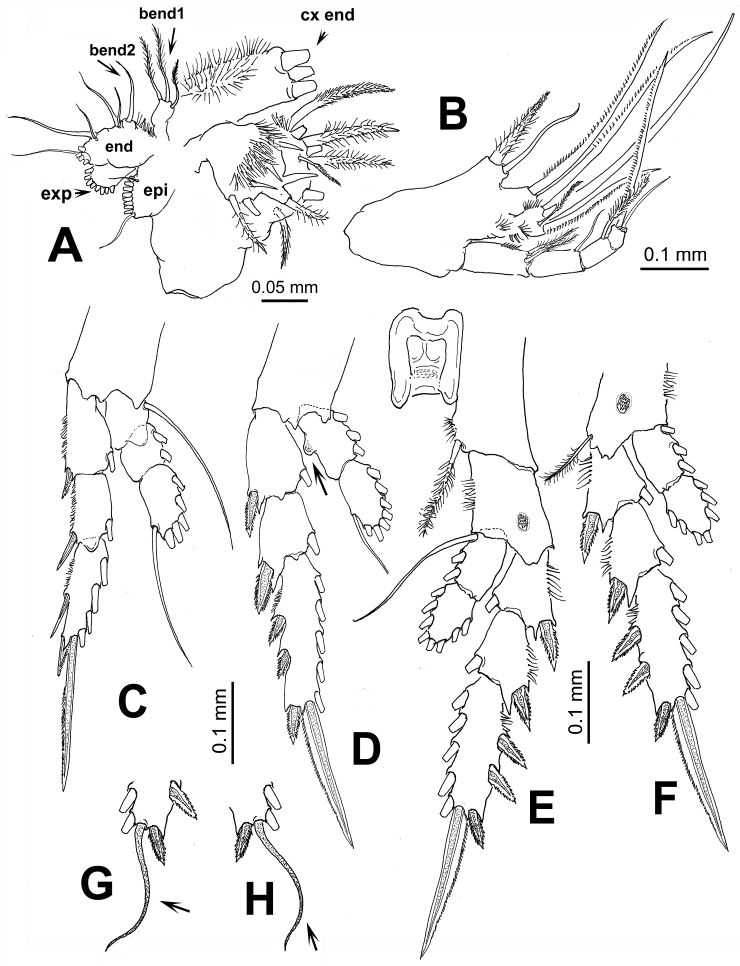
*Pontellopsis lubbockii* (Giesbrecht) from the Mexican Pacific. Adult female **A** maxillule showing armature of coxal endite (**cx end** distal spiniform elements cut short), proximal basal endite (**bend1**), distal basal endite (**bend2**), epipodite (**epi**), exopod (**exp**), and endopod (**end**) **B** maxilliped **C** leg 1 **D** leg 2 **E** eg 3 **F** leg 4 **G** variant form of leg 3 third exopodal segment with flexible terminal setal element (arrowed) **H** same, leg 4.

Maxillule (Fig. 3A) typical of pontellids, praecoxal arthrite with 14 setal elements; coxal endite (cx end) with 3 long, robust spine-like elements on endite and 9 setae on epipodite (epi); basis with 3 and 1 setae on proximal (bend1) and distal (bend2) endites, respectively; 1st and 2nd endopod segments, each with 2 setae, incorporated into basis, distal endopod segment with 5 apical setae; exopod with 8 setae.

Maxilla (Fig. 2F) uniramous, first praecoxal endite bearing 4 setae, second with 3 setae (one of them shorter and thinner than the others); two coxal endites each bearing 3 setae. Basis with 2 setae; endopod 4-segmented, setal formula of endopod as: 2, 2, 1, 1. Basal and endopodal setae strongly serrate.

Maxilliped (Fig. 3B) uniramous, with praecoxa and coxa fused, three syncoxal endites well developed, with setal formula 2, 2, 3; endites setae strong, serrate. Inner lateral margin of third endite with rows of short setae. Basis fringed with medial row of 5-6 spinules and 2 distal setae. Endopod 4-segmented, setal formula of endopod as: 2, 1, 1, 2.

Leg 1 with 3-segmented endopod; legs 2-4 with 2-segmented endopods and 3-segmented exopods (Figs 3C-F). Coxae with plumose inner seta; basis of leg 4 with slender outer seta, medial patch of spinules on medial anterior margin of legs 3 and 4. First endopodal segment of second leg with inner rounded protuberance (arrowed in Fig. 3D). In one specimen examined, terminal exopodal spine of legs 3 and 4 modified, represented by flexible seta (Italized in setal formula) (Fig. 3G, H). Seta and spine formula (Arabic numbers=setae, Roman numerals=spines) of legs 1-4 as:

 Coxa Basis Exopod Endopod

Leg 1 0-1 0-0 I-1;I-1;II,I,4 0-1;0-2;1,2,3

Leg 2 0-1 0-0 I-1; I-1;III,I,5 0-3; 2,2,4

Leg 3 0-1 0-0 I-1; I-1; III,*1*,5 0-3; 2,2,4

Leg 4 0-1 1-0 I-1; I-1;III,*1*,5 0-3; 2,2,3

Leg 5 (Fig. 1I, J) biramous, slightly asymmetrical; coxa and intercoxal sclerite fused. Basis subrectangular, naked. Endopod distally bifurcate, about 0.3 times as long as exopodal ramus. Exopod of both legs 1-segmented, elongate, right leg with 3 outer spiniform processes and a large distal inner process; left leg smooth except for two subdistal outer spine-like setae.

##### Male.

**Figure 4. F4:**
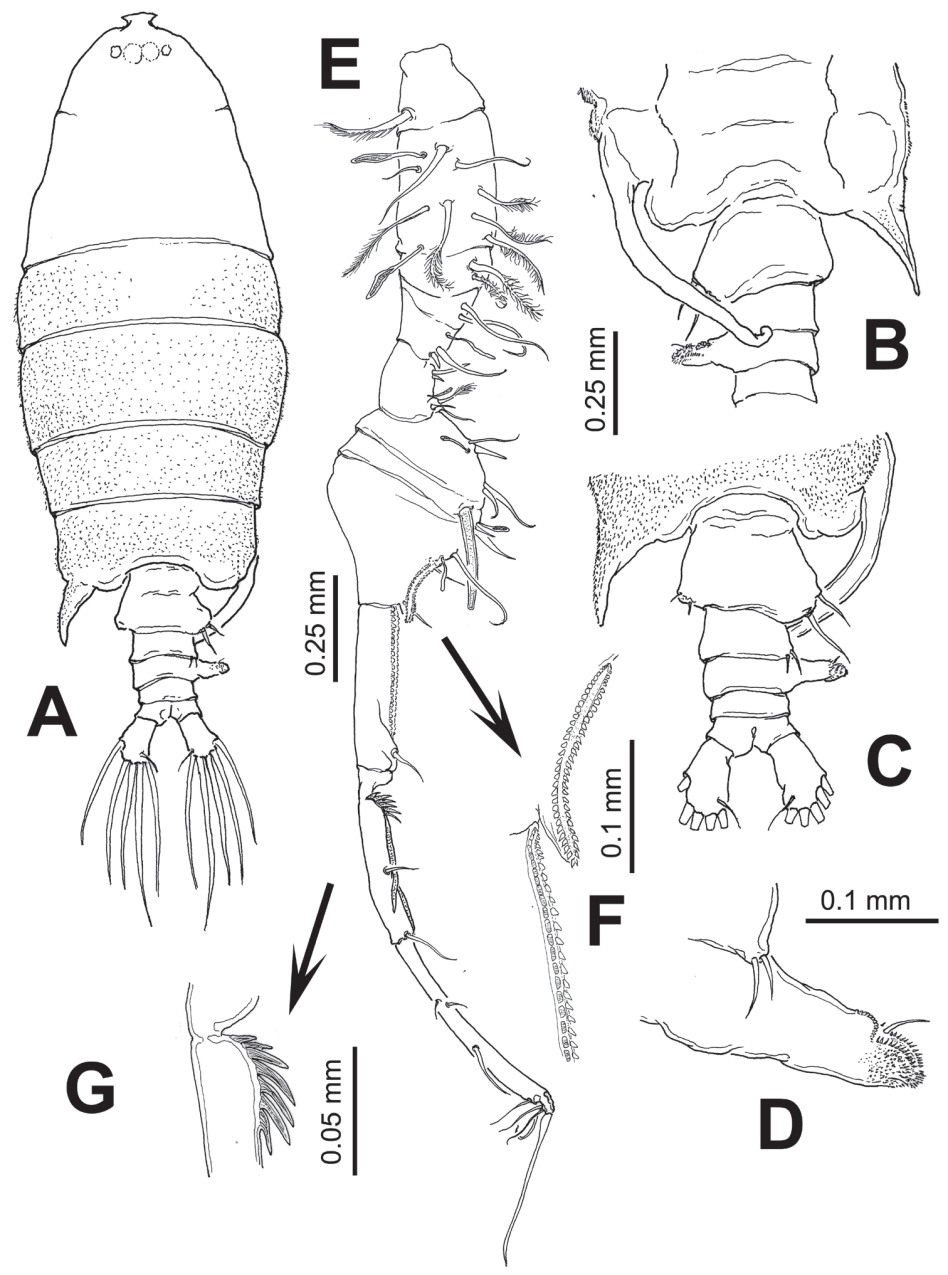
*Pontellopsis lubbockii* (Giesbrecht) from the Mexican Pacific. Adult male **A** habitus, dorsal view **B** urosome, ventral view **C** same, dorsal view **D** detail of process on right margin of third urosomite **E** geniculate antennule **F** detail of ornamentation on antennular segments 9 and 10 (arrowed) **G** detail of ornamentation of proximal part of antennular segment 11 (arrowed).

Body (Fig. 4A) robust, slightly smaller than female (1.85–2.07 mm, average: 1.98 mm, *n*=4). Cephalosome about 3.5 times as long as urosome (caudal rami excluded), dorsal surface of cephalosome pilose, particularly pedigerous somites 1-5. Fifth pedigerous somite with asymmetrical lateral expansions, left process spiniform, reaching posterior margin of first urosomite; right side with long curved, ventromedially directed process with small, distally curved rounded process (Fig. 4B). Urosome (Fig. 4A-C) with 5 somites. Genital double-somite strongly asymmetrical, left side with 2 sensilla on outer distal corner; right side expanded forming rounded process armed with two unequal setae (Fig. 4C). Second urosomite with pair of sensillae on right side; third urosomite as long as succeeding somite, with strong laterally-directed rod-like process on right margin, process armed with anterodistal curved row of teeth-like spinules, a short seta, and terminal rows of spinules (Fig. 4D). Anal somite symmetrical, as long as preceding somite. Caudal rami slightly asymmetrical, approximately twice as long as wide.

**Figure 5.**
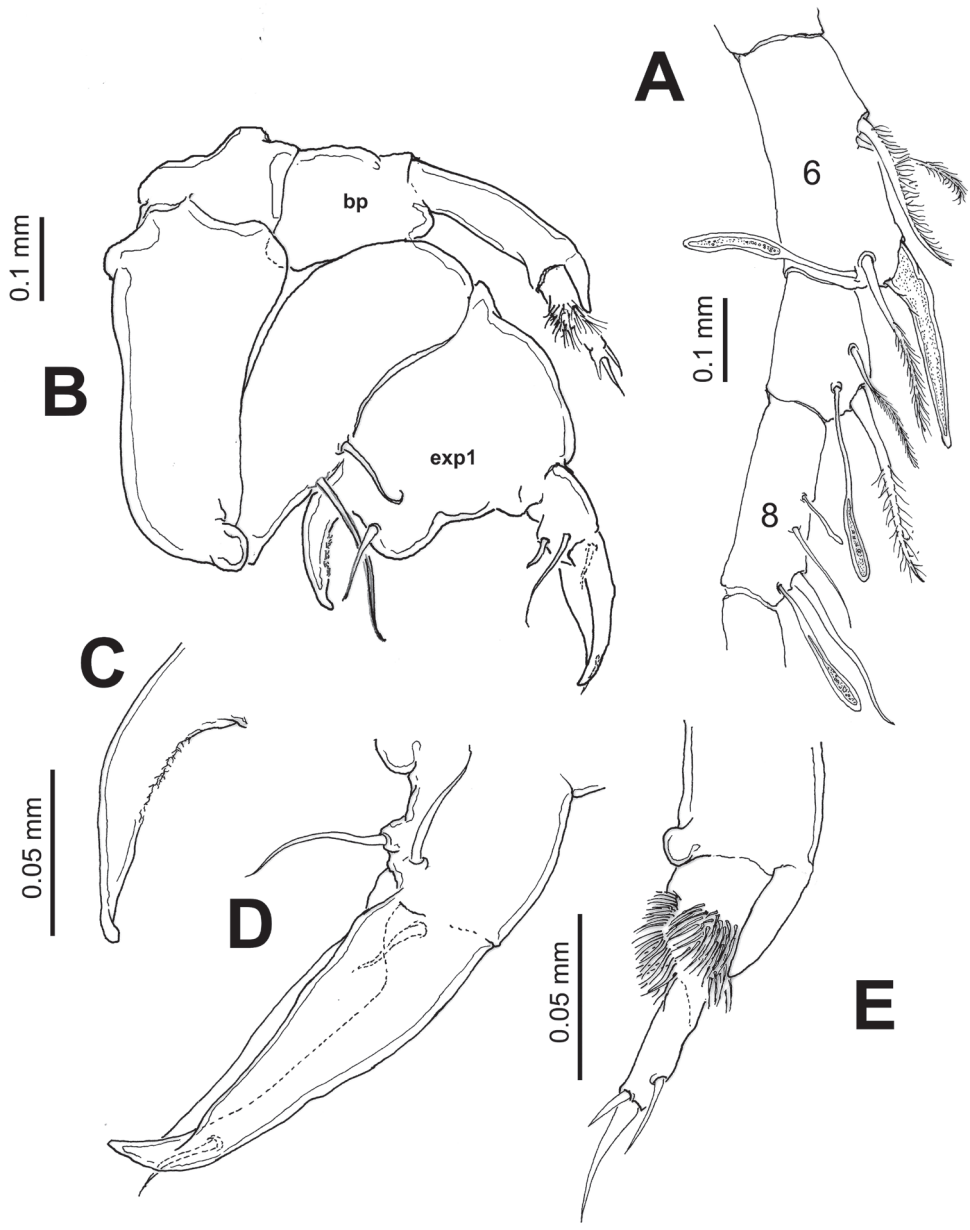
*Pontellopsis lubbockii* (Giesbrecht) from the Mexican Pacific. Adult male **A** left antennule, segments 6–8 showing spiniform process on segment 6 **B**. leg 5 showing basipod (**bp**) of left ramus and first exopodal segment of right ramus (**exp1**) **C** right leg, detail of basal thumb of chela **D** right leg, detail of second exopodal segment or distal finger of chela **E** left leg, distal segments and ornamentation.

Right antennule (Fig. 4E–G) with 12 segments geniculate between segments 10–11, reaching middle of third pedigerous somite. Antennular segments armed as follows (Arabic numbers= setae; Roman numerals= spines, aes=aesthetascs): 1 (I-III) (1), 2 (IV-VII) (8+2aes), 3 (VIII-X) (2), 4 (?) (2), 5 (?) (2+aes), 6 (X-XIV) (5+ 2aes), 7 (XV-XVI) (4+aes), 8 (XVII) (2,I+aes), 9 (XVIII-XIX) (3+aes), 10 (XX) (1), 11(XXI-XXIII) (1,II), 12 (XXIV-XXVIII) (8+aes). Spine on segment 8 long, slightly curved; segments 9 and 10 with coarse double row of acuminate sharp teeth (Fig. 4F). Segment 11 with proximal process forming fan-like row of strong spines plus two usual stout spines adjacent to segmental margin (Fig. 4G). Anterior margin of segments 10 and 11 with usual spiniform processes parallel to segmental margin. Left antennule as in female except for shorter spiniform process on segment 6 which is also relatively shorter than in female (Fig. 5A).

Leg 5 (Figs 5B–E) asymmetrical, typical of pontellids. Left leg 5 short; coxa quadrate, basipod (bp) robust, cylindrical, naked. Exopod 3-segmented, segments 2–3 partly fused; first segment cylindrical, with subtriangular process on outer distal margin. Second exopodal segment (Fig. 5E) with medial surface covered by patch of long hair-like setae, segment with inner rounded expansion and subdistal seta on outer lateral margin; third segment with 2 unequal spines plus inner spiniform process. Right leg 5 basis with 2 unequal setae. Exopod with two segments, forming robust, widely open chela; first segment (exp1) forming thumb of chela ending in short, strong process curving inward with inner surface armed with shallow cuticular ridges and small spinules (Fig. 5C). Second exopodal segment forming distal elongate finger, tapering distally, armed with two subequal proximal setae on outer surface plus one proximal and one distal setae inserted on inner surface of segment (Fig. 5D).

##### Remarks.

Our specimens from the Mexican Pacific were identified as *Pontellopsis lubbockii* by the females having acute, symmetrical posterolateral corners of the fifth pedigerous somite plus an asymmetrical genital double-somite as long as the anal somite and with two dorsal protuberances. Males have a long, curved process on the right side of the fifth pedigerous somite, a laterally directed process on the third urosomite combined with a pair of long stout setae on the right margin of the genital double somite. Females of this species are easily distinguishable from its congeners by the structure and details of the genital double somite. It is unique in having two conical dorsal processes and also a ventral spine arising from the genital field. One of these processes might have been overlooked in previous descriptions (Giesbrecht 1889; Pillai 1977) but its presence was confirmed in museum specimens from California (USNM-109384) and off Ecuador (USNM-80382). There are other species of *Pontellopsis* bearing dorsal processes, like *Pontellopsis inflatodigitata* Chen & Shen, 1974, *Pontellopsis laminata* Wilson, 1950, *Pontellopsis herdmani* Thompson & Scott, 1903, *Pontellopsis scotti* Sewell, 1932, *Pontellopsis macronyx* Scott, 1909, and *Pontellopsis yamadae* Mori, 1937. Only one such dorsal process is illustrated in previous illustrations of *Pontellopsis lubbockii*, appearing as a single, robust, mammiliform, dorsal process (Giesbrecht 1893; Pillai 1977), but our redescription shows that there are two conspicuous processes; a similar pattern is present in *Pontellopsis albatrossi* Wilson, 1950. When two dorsal processes are present, they are differently built; in *Pontellopsis laminata*, the left process is very large, clearly spiniform, laterally projected, whereas the right one is reduced to a low protuberance (see Pillai 1977). In *Pontellopsis herdmani*, there are two thorn-like projections on the left side (Thompson and Scott 1903); *Pontellopsis lubbockii* also differs from *Pontellopsis scotti*, which has a single spiniform dorsolateral process and an enlarged right proximal spine. *Pontellopsis macronyx* has a pair of dorsal spiniform processes, different from the robust, conical processes found in *Pontellopsis lubbockii* (Scott 1909; Chen and Shen 1974). A different pattern, with a single globose lateral process tapering distally into a spine was depicted for the same nominal species by Silas and Pillai (1973), but it also diverges from the pattern observed in *Pontellopsis lubbockii*. The structure of the female genital double-somite of *Pontellopsis yamadae* is probably the most similar to that of *Pontellopsis lubbockii* and in some cases both species may be confused, but the dorsal processes are quite distinct, digitiform, none of them reaching the dorsal margin of the somite (Mori 1937; Jeong et al. 2009). Both species also differ in the structure of the thoracic processes, short, rounded in *Pontellopsis yamadae* and long, spiniform in *Pontellopsis lubbockii*. The structure of the female leg 5 is also different in both species, with a much shorter and more robust outer ramus in *Pontellopsis yamadae* (see Mori 1937).

The extremely long spiniform ventral process present in the genital double-somite of *Pontellopsis lubbockii*, is a unique character of this species and has not been hitherto described in or illustrated in previous works (Giesbrecht 1893; Pillai 1977). In only a few species of the genus a ventral process related to the genital field has been described: in *Pontellopsis albatrossi*, *Pontellopsis armata*, and *Pontellopsis villosa* (Brady, 1883) it is a short, curved spine arising from the genital field (Zheng et al. 1982). Yet another interesting character of *Pontellopsis lubbockii* is the modification of the distal spines of the third exopodal segment of legs 3 and 4, they are flexible elements, thus contrasting with the usual pattern of stout, spiniform terminal setae. The data available to us from various descriptions suggest that this is a unique character among members of this genus.

The mandibular dentition found in our specimens agrees with the pattern described by Fleminger (1956) for this species and genus; dentition is quite uniform among species of *Pontellopsis* and its taxonomical value is weak. In addition, this species has the main characters described by Ohtsuka and Onbé (1991) as Type II specialized mouthparts for predation, with serrate maxillar setae, a relatively narrow mandibular edge armed with sharp, blade-like teeth, and clusters of setae and spinules near the base of the teeth. Overall, our analysis supports the notion that this species is a predator, as long known for other species of *Pontellopsis* (Lillelund and Lasker 1971).

### Distribution of *Pontellopsis* in the Eastern Tropical Pacific

In the Eastern Pacific, particularly in the California Current region, only a few species of *Pontellopsis* have been recorded: *Pontellopsis occidentalis* Esterly, 1906, *Pontellopsis regalis* (Dana, 1849), and *Pontellopsis lubbockii*. *Pontellopsis occidentalis* is regarded as endemic of southern California, the Gulf of California, and Baja California area. *Pontellopsis regalis* is frequently found in waters of the ETP (Fleminger 1967a; Brinton et al. 1986; Hernández-Trujillo 1989, 1994). Additional records of the genus are found south of the California Current region, off the southern sector of Baja California and the Mexican Pacific: *Pontellopsis armata* (Giesbrecht, 1889), *Pontellopsis tenuicauda* (Giesbrecht, 1889), *Pontellopsis brevis* (Giesbrecht, 1889), *Pontellopsis perspicax* (Dana, 1849), and *Pontellopsis yamadae* Mori, 1937 (Hernández-Trujillo 1989, 1994; Suárez-Morales and Gasca 1998; Palomares-García et al. 1998; Hernández-Trujillo et al. 2004). So far, only 8 out of the 33 known species of the genus have been recorded in the Eastern Tropical Pacific. The genus is clearly more diverse in the Indo-West Pacific, a region harboring many endemic or presumably endemic forms as a result of the geological history and biogeographic processes related to that geologically complex area (Fleminger 1986).

### Key to the species of *Pontellopsis* of the Eastern Pacific

#### Females

**Table:** 

1A	Posterolateral corners of fifth pedigerous somite with terminally rounded processes (Figs 6A, E)	2
1B	Posterolateral corners of fifth pedigerous somite forming acute spiniform processes (Fig. 6B, C, D, F)	3
2A	Genital double-somite elongate, with 2 acute dorsal processes of unequal size on posterior half of somite (Fig. 6E)	*Pontellopsis yamadae*
2B	Genital double-somite with 2 unequal spiniform processes, one small, one long, in right side of posterior half of somite (Fig. 6A)	*Pontellopsis tenuicauda*
3A	Spiniform processes of fifth pedigerous somite reaching the middle length of anal somite or beyond (Fig. 6C, D)	4
3B	Spiniform processes of fifth pedigerous somite not as long, barely reaching the posterior margin of the genital double-somite or even shorter (Fig. 6 B, F)	5
4A	Genital double-somite with strong, thumb-like process on left margin. Anal somite half the length of genital double-somite (Fig. 6D)	*Pontellopsis villosa*
4B	Genital double-somite without distinct process. Anal somite as long as genital double-somite (Fig. 6C)	*Pontellopsis armata*
5A	Genital double-somite as long as or slightly longer than anal somite, with processes or expansions on both margins or on dorsal surface	6
5B	Genital double-somite twice as long as anal somite, with lateral process on right margin only	*Pontellopsis occidentalis*
6A	Genital double- somite with two dorsal conical unequal protuberances	*Pontellopsis lubbockii*
6	BGenital double- somite with no such dorsal processes	7
7A	Both lateral margins of genital double-somite expanded forming nearly symmetrical rounded processes, that on the right side globular; anal somite strongly produced between caudal rami (Fig. 6B)	*Pontellopsis perspicax*
7B	Genital double-somite with asymmetrical, rounded lateral processes, anal somite not strongly produced between caudal rami (Fig. 6F)	*Pontellopsis regalis*

#### Males

**Table:** 

1A	Posterolateral corners of fifth pedigerous somite with symmetrical or nearly symmetrical processes (Fig. 6G)	2
1B	Posterolateral corners of fifth pedigerous somite with strongly asymmetrical processes, with long, slender, curved process on the right side (Fig. 6H)	3
2A	Second urosomite with small lateral process on the left margin; second exopodal segment of left leg 5 cylindrical, as long as preceding segment (Fig. 6O), process on first exopodal segment of right leg 5 very short, distally blunt	*Pontellopsis occidentalis*
2B	Second urosomite without such process on left margin; second exopodal segment of left leg 5 globose, half as long as preceding segment (Fig. 6P), process on first exopodal segment of right leg 5 short, distally acute	*Pontellopsis villosa*
3A	Left posterolateral corner of fifth pedigerous somite forming short terminally rounded or broadly subtriangular process, not acute (Fig. 6I)	4
3B	Left posterolateral corner of fifth pedigerous somite forming relatively long acute process (Fig. 6H)	5
4A	Second and third urosomites with weak lateral expansions (Fig. 6I), process on first exopodal segment of right leg 5 long, distally truncate (arrow in Fig. 6L)	*Pontellopsis tenuicauda*
4B	Second and/or third urosomites with lateral expansion on right side, process on first exopodal segment of right leg 5 long, tapering distally (Fig. 6K, M, N)	7
5A	First urosomite symmetrical, armed with small unequal setae inserted on posterolateral margin	6
5B	First urosomite clearly asymmetrical, with rounded process on right lateral margin; process armed with two long, stout setae	*Pontellopsis lubbockii*
6A	Right posterolateral corner of fifth pedigerous somite long, acute, tapering distally (Fig. 6I); caudal rami as long as wide, distal segment of chela with protuberance on medial position of inner margin (arrow Fig. 6K)	*Pontellopsis regalis*
6B	Right posterolateral corner of fifth pedigerous somite long, slender from insertion, branch-like (Fig. 6H); caudal rami twice as long as wide, distal segment of chela with low proximal expansion on inner margin	*Pontellopsis armata*
7A	Second and third urosomites expanded laterally, process on first exopodal segment of right leg 5 shorter than second exopodal segment (Fig. 6N)	*Pontellopsis yamadae*
7B	Only third urosomite expanded laterally. Right leg 5 with finger-like process of first exopodal segment longer than second exopodal segment (Fig. 6M)	*Pontellopsis perspicax*

**Figure 6.**
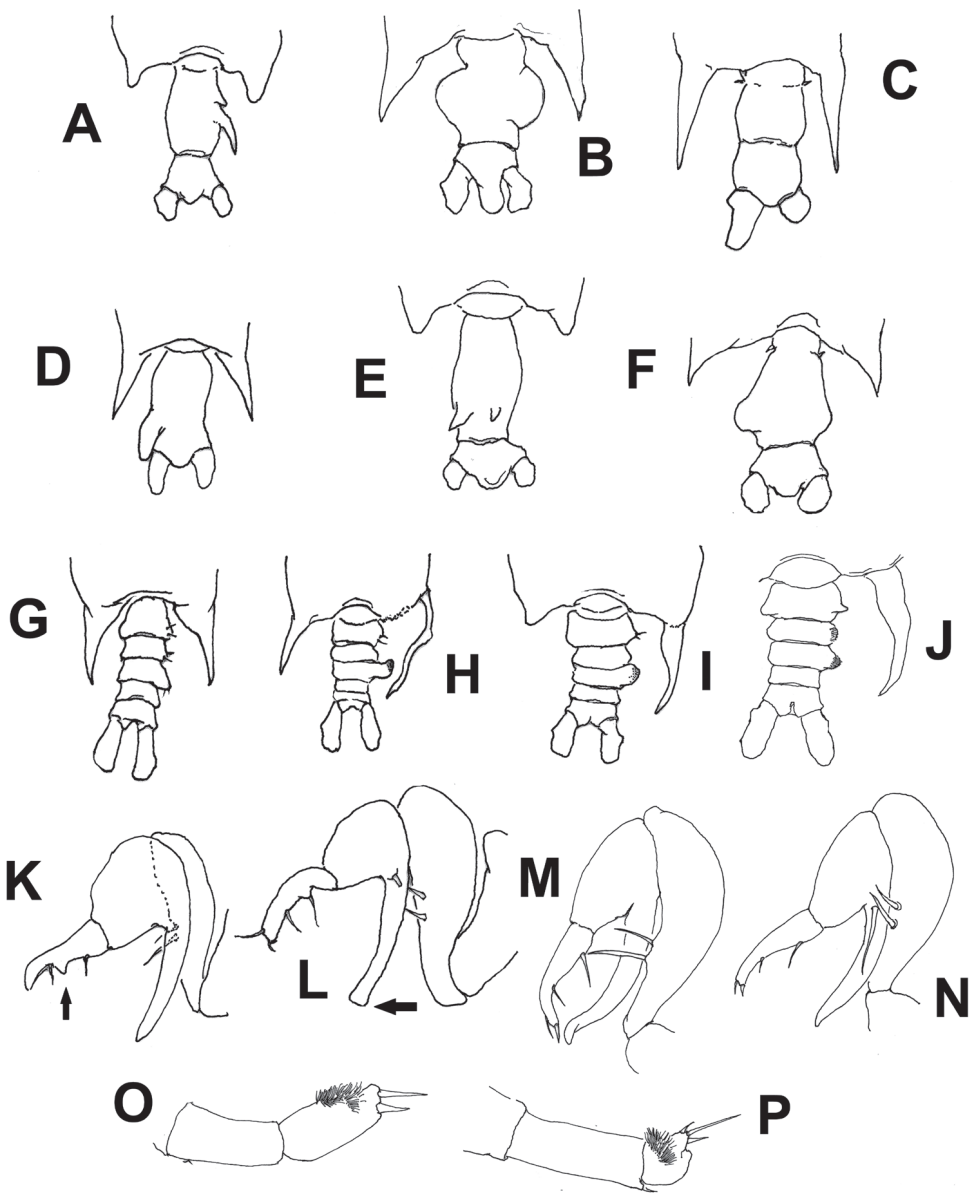
Schematic illustrations of characters used in the identification key to species of *Pontellopsis* from the Eastern Tropical Pacific. Explanation in key couplets. Illustrations modified from Giesbrecht (1893), Mori (1937), Chen and Zheng (1965), Mulyadi (2002), and Palomares-García et al. (1998).

## Supplementary Material

XML Treatment for Monops lubbockii
